# Thymoquinone as a Potential Adjuvant Therapy for Cancer Treatment: Evidence from Preclinical Studies

**DOI:** 10.3389/fphar.2017.00295

**Published:** 2017-06-12

**Authors:** A.G.M. Mostofa, Md Kamal Hossain, Debasish Basak, Muhammad Shahdaat Bin Sayeed

**Affiliations:** ^1^Department of Clinical Pharmacy and Pharmacology, University of DhakaDhaka, Bangladesh; ^2^Department of Pharmaceutical Chemistry, University of DhakaDhaka, Bangladesh

**Keywords:** thymoquinone, cancer treatment, adjuvant therapy, preclinical studies

## Abstract

Thymoquinone (TQ), the main bioactive component of Nigella sativa, has been found to exhibit anticancer effects in numerous preclinical studies. Due to its multitargeting nature, TQ interferes in a wide range of tumorigenic processes and counteracts carcinogenesis, malignant growth, invasion, migration, and angiogenesis. Moreover, TQ can specifically sensitize tumor cells toward conventional cancer treatments (e.g., radiotherapy, chemotherapy, and immunotherapy) and simultaneously minimize therapy-associated toxic effects in normal cells. In this review, we summarized the adjuvant potential of TQ as observed in various *in vitro* and *in vivo* animal models and discussed the pharmacological properties of TQ to rationalize its supplementary role in potentiating the efficacy of standard therapeutic modalities namely surgery, radiotherapy, chemotherapy, and immunotherapy. Altogether, we suggest further comprehensive evaluation of TQ in preclinical and clinical levels to delineate its implied utility as a novel complementary adjuvant therapy for cancer treatment.

## Introduction

For the last five decades, large-scale “war on cancer” leads to significant progress in the development of new therapeutic options and more in-depth insights about various malignancies. Despite the emergence of numerous treatment strategies such as chemotherapeutic agents, small molecule inhibitors, specific gene, or protein targeting so-called smart drugs, and immunotherapies that are found to be effective in the treatment of some form of cancers (e.g., childhood leukemia, human epidermal growth factor receptor 2-positive breast cancer), cancer death rate, in general, has not changed substantially (only 5% since 1950; Sung et al., 2012). According to Centers for Disease control and Prevention (CDC), cancer is the second leading cause of death in US (591,699 deaths registered in 2015–2016), almost catching up heart diseases (https://www.cdc.gov/nchs/fastats/leading-causes-of-death.htm). Several underlying factors have been identified for failures of current cancer therapies, and among these factors, intratumoral heterogeneity is the most prominent. Targeting a particular gene, gene product, or signaling pathway can only eliminate a specific group of cells from the tumor, other genetically distinct variants can easily escape from that treatment and start developing tumor at surrounding area or may metastasize to distant sites. Due to this fact, most cancer treatment involves combination therapies where each drug works by different mechanisms and thus diminishing the chance of developing resistance.

**Figure 1:**
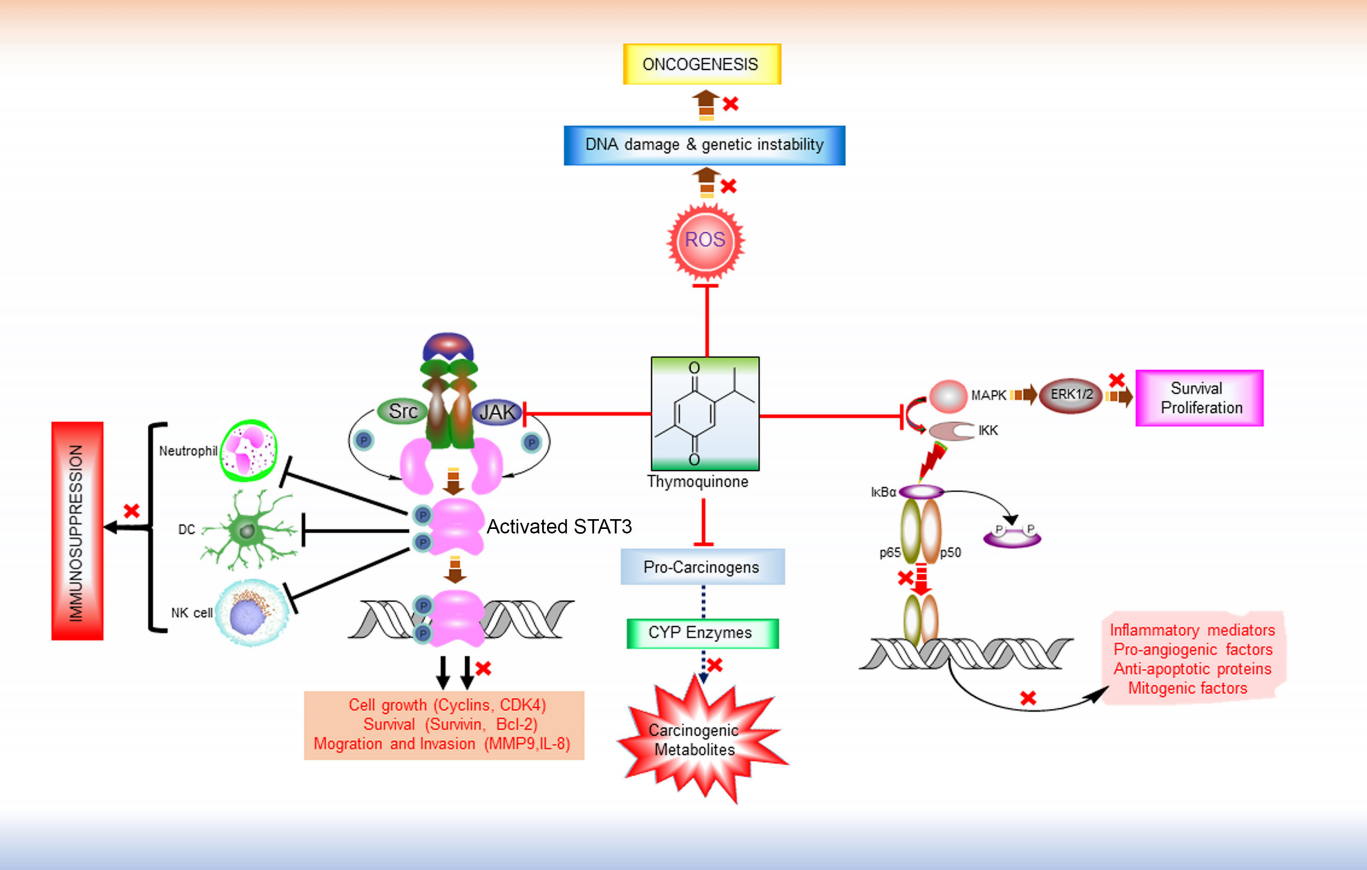
**Major Anti-tumorigenic pharmacological properties of thymoquinone**. TQ is a multi-targeting anticancer molecule. It prevents the formation of carcinogenic metabolites from pro-carcinogens by inhibiting CYP enzymes. By virtue of its free radical scavenging properties, TQ can inhibit ROS-mediated DNA damage induction and genetic instability and thereby prevent tumorigenesis. TQ also exhibits anti-proliferative and anti-survival effects by interfering in the MAPK/ERK pathway. In addition, TQ is found to modulate the activity of various transcription factors like NF-κB and STAT3. IKKs activates NF-κB by inducing the phosphorylation and proteasomal degradation of NF-κB inhibitor IκBa. The resulting free NF-κB (e.g., heterodimer of p65 and p50 subunits) then translocates to the nucleus and activate the transcription of various target genes that encodes numerous inflammatory mediators, pro-angiogenic factors, anti-apoptotic proteins. By targeting IKKs and thus inhibiting NF-κB activation, TQ shows anti-inflammatory, anti-angiogenic, and pro-apoptotic effects. Persistent STAT3 activity is a common feature in various malignancies. Overactive STAT3 leads to the dysregulation of immune response in tumor microenvironment by interfering in the proliferation and activation of various immune cells (e.g., NK cells, Neutrophils), maturation of DC, activation of tumor-antigen-specific CD8+ T cells, and differentiation of plasma cells. TQ has shown inhibitory effect on both the constitutive and ligand-induced activation of these transcription factors by disrupting their upstream signaling pathways, and thereby might reverse immune suppression and potentiate the efficacy of immunotherapeutic agents. ROS, Reactive oxygen species; MAPK, Mitogen-activated protein kinase; ERK 1/2, extracellular signal-regulated protein kinase 1 and 2; IKK, IκB kinases; Src, Non-receptor tyrosine kinase (Sarcoma-family kinases); JAK, Janus activated kinase; STAT3, Signal transducer and activator of transcription 3; DC, Dendritic cell; NK Cell, Natural killer cells.

Thymoquinone (2-methyl-5-isopropyl-1, 4-benzoquinone; TQ), a monoterpene, is the main active ingredient of the volatile oil of *Nigella sativa* L. (NS) (family Renunculaceae) which is familiar as black cumin or black seed (Salomi et al., 1991). Black seed is traditionally used both as condiment and natural medicine in many societies including Indian subcontinents and Arabian countries. Ayurvedic and Unani medicinal systems have been recommending to use black seed oil for the treatment of various human diseases such as bronchial asthma, dysentery, headache, gastrointestinal problems, eczema, hypertension, and obesity. Some recent clinical studies have found potent anti-inflammatory and antioxidant effects of oral NS extracts (Dehkordi and Kamkhah, 2008; Al-Jenoobi et al., 2010). Hence, the usefulness of black cumin seed in cancer prevention and treatment is now more than a speculation. TQ is first isolated from NS extracts in 1963 by El-Dakhakhany, since then diverse pharmacological properties of TQ have been reported from various studies (Salomi et al., 1991; Dajani et al., 2016). Numerous preclinical studies have been performed to determine the anticancer effects of TQ where it has exhibited selective cytotoxicity for human cancer cells (Gali-Muhtasib et al., 2004). In addition to its cell death and tumor growth inhibitory activities, TQ is found to interfere with other tumorigenic processes including angiogenesis, invasion, and metastasis (Peng et al., 2013; Khan et al., 2015). Furthermore, TQ can sensitize cancer cells to conventional chemotherapy and radiotherapy by modulating the resistance mechanisms (Velho-Pereira et al., 2011; Zhang et al., 2016). Multiple targets (e.g., carcinogen metabolizing enzymes, transcription factors, cell cycle regulatory proteins, etc.) of TQ have been identified that are somehow involved in tumorigenesis or development of drug resistance (Kundu et al., 2014b) (Figure 1). Though treatment with TQ alone has shown antitumor efficacy in several *in vitro* and *in vivo* studies as mentioned in details in a recent review (Majdalawieh et al., 2017), the lower efficacy (Effenberger et al., 2010) and poor bioavailability (Ganea et al., 2010; Elmowafy et al., 2016) of TQ is the primary bottleneck for considering it as the primary therapeutic agent. Therefore, in this review, we proposed the potential role of TQ as an adjuvant therapy with different types of conventional cancer treatments namely surgery, radiotherapy, chemotherapy, and immunotherapy either to prevent carcinogenesis or to potentiate the efficiency of conventional therapeutic modalities.

## Pharmacological properties of TQ indicates its potential role as an adjuvant therapy for surgery

Though surgical tumor removal remains the first option for most malignancies (~60% of all cancer patients have to go through surgical procedures; Demicheli et al., 2008), many cancers reappear after surgery and start proliferating more aggressively. Another unavoidable consequence of surgical trauma is induction of metastatic potential in the microscopic tumors that are not eliminated through tumor resection (Ceelen et al., 2007, 2014). Numerous mitogenic and pro-angiogenic factors, such as transforming growth factor beta (TGF-β), fibroblast growth factor (FGF), vascular endothelial growth factor (VEGF), epidermal growth factor (EGF)-like growth factors, endostatin etc., were found in the wound fluids which explains the stimulation of cancer cell proliferation and neoangiogenesis during post-surgery wound healing period (Maniwa et al., 1998; Abramovitch et al., 1999; Wu et al., 2003). Therefore, several pre-operative and post-operative adjuvant therapies are administered in order to shrink the tumor into an operable stage and to prevent spreading of cancer cells from primary tumor sites. Post-operative therapies are important to eradicate the residual cancer cells (microscopic circulating tumor cells escaped from surgery) and to counteract the surgical trauma-induced pro-angiogenic and metastatic signaling pathways. Unfortunately, the current neoadjuvant therapies (mostly radiation and chemotherapy) are unable to elicit such ideal therapeutic efficacies and in many cases deteriorate patient quality of life due to unavoidable adverse effects. Therefore, novel multi-targeting, safe, and effective neoadjuvant treatments are urgently needed to improve the present standard of surgical tumor resection.

Treatment with TQ showed significant attenuation of tumorigenic signaling, including those mediated by TGF-β, VEGF, FGF, EGF, and several other pro-mitogenic, angiogenic, and metastatic factors, with a consequent dose-dependent inhibition of cancer cell growth, migration, and invasion (Yi et al., 2008; Ahmad et al., 2013; Khan et al., 2015; Rajput et al., 2015). It has also been reported that TQ can counteract the trauma-induced chemotaxis of circulating malignant cells and their epithelial to mesenchymal transition (EMT; Badr et al., 2011b; Ahmad et al., 2013; Khan et al., 2015). Moreover, TQ is found to interfere in the activation of various transcription factors including nuclear factor erythroid-related factor-2 (Nrf-2), nuclear factor-kappaB (NF-κB), signal transducer and activator of transcription-3 (STAT-3) that are responsible for the transcriptional activation of genes encoding proteins involved in cell proliferation, angiogenesis, and metastasis (Kundu et al., 2014c; Zhang et al., 2016). Overall, TQ has favorable pharmacological properties to counteract surgery-associated tumor invasion and metastasis. Although no direct study has been conducted to determine the usefulness of TQ along with cancer surgery, emerging evidences warrant preclinical and clinical evaluation of TQ as a pre-operative and/or post-operative neoadjuvant therapy.

## TQ with a dual mode of action: radioprotection and radiosensitization

Radiotherapy is the most widely utilized therapeutic modality in cancer management, almost 50% of all cancer patients receive this therapy in one form or another during their course of illness (Delaney et al., 2005). In many cases when tumors are inoperable, radiation is the main alternative to restrain the disease progression. Though radiation therapy is highly effective in tumor cell eradication, its success in cancer treatment is restricted by some inherent limitations, particularly the deleterious effect to surrounding normal tissues and stimulation of cancer cells adaptive responses to counteract the damage process. Since radiotherapy is generally applied in a course of multiple fractions, cancer cells that survived after initial cycles acquire resistance through multiple cellular mechanisms including activation of NF-κB, phosphatidylinositol 3-kinase (PI3K), protein kinase B (Akt), mammalian target of rapamycin (mTOR), and become less responsive to the later cycles of radiotherapy and/or chemotherapy (Baskar et al., 2014). Higher doses of ionizing radiation can effectively kill all tumor cells irrespective of cancer types and resistance status; however, application of such radiation beam may severely impact on normal cells. Therefore, current efforts in radiation research have aimed to devise strategies that will make tumor tissue more sensitive to ionizing radiation while protecting normal cells.

To maximize the benefit, radiotherapy is often supplemented with either radiosensitizers to intensify the radiation-induced cytotoxicity through augmenting biomolecular damage (e.g., DNA damaging agents) or radioprotectors to mitigate the deleterious effect of ionizing radiation in normal tissues mostly by scavenging highly reactive free radicals (e.g., antioxidants). Currently available radiosensitizers are unable to exhibit tumor cell specific radiation sensitization unless administered through targeted delivery. Similarly, radioprotectors inextricably scavenge the ionizing radiation-induced reactive free radicals and interfere in the efficacy of radiotherapy. Therefore, we need an adjuvant therapy that can simultaneously exert radiosensitization in cancer cells and radioprotection in healthy cells. In this regard, TQ can be an ideal candidate. Reelma et al. confirmed that TQ can enhance the cytotoxic efficacy of radiation through modulation of cell cycle and apoptosis (Velho-Pereira et al., 2011). Another study reported the prevention of radiation-induced metastatic progression of breast cancer cells by TQ through restoration of TGF-β (Rajput et al., 2015). Moreover, it has been found that TQ can directly modulate the activation of several signal transduction pathways including PI3K-Akt-mTOR that are frequently upregulated in various cancers and confer resistance to radiotherapy (Baskar et al., 2014; Kundu et al., 2014b). Radio-protective effect of TQ or *Nigella sativa* seed extracts was also observed in some *in vivo* studies (Velho-Pereira et al., 2012; Orhon et al., 2016). Results from those studies showed that animal treated with macerated extracts of *Nigella sativa* L. seeds (contains TQ along with other components) experienced less damage in their liver, spleen, brain, and intestines upon exposure to radiation. Another recently published work demonstrated the rescue of T-lymphocytes by TQ from gamma irradiation-induced apoptosis (Guida et al., 2016). From mechanistic point of view, these radioprotective effect of TQ is mainly mediated through its free radical scavenging ability and anti-oxidant properties (Velho-Pereira et al., 2012). Collectively, TQ can be a suitable adjuvant therapy for radiation treatment of cancer. Further studies are required to determine the differential dose and treatment schedule of TQ and radiation to achieve the best therapeutic outcome.

## Chemo-potentiating role of TQ

A wide variety of chemotherapeutic drugs (more than 100) have been used in the treatment of different malignancies since 1940s. Despite the advent of numerous highly efficient cytotoxic agents, the overall rate of cancer-related death barely reduced in these 70 years of extensive researches and development. Some major drawbacks of currently used anticancer chemotherapy include non-specific cytotoxicity which results in bone marrow suppression and other organ toxicity and development of drug resistance by cancer cells. An augmented drug efflux, alteration of the molecular target, increased repair of drug-induced DNA damage, activation/suppression of signaling pathways leading to an upregulation of survival molecules and avoidance of apoptosis have been reported to be the most prominent reasons of tumor cell recalcitrance against chemotherapy (Baguley, 2010; Zahreddine and Borden, 2013; Alfarouk et al., 2015). Furthermore, heterogeneous nature of cancer cells is another hurdle that curbs the efficacy of targeted drug delivery or aiming a particular cancer-specific tumorigenic pathways. Hence, a suitable adjuvant along with the novel treatment approaches is obviously the Holy Grail for cancer patients.

There are ample evidences (mostly preclinical studies) showing that TQ, alone or in combination with other main course chemotherapeutic agents, can significantly impede cancer progression and synergistically reduce tumor burden in various malignancies through alteration of multiple tumorigenic pathways (Table 1). The administration of *Nigella sativa* seed extract enriched with TQ suppressed mice skin papillomagenesis when applied topicall (Salomi et al., 1991). In a similar fashion, 7,12-dimethylbenz[a]anthracene DMBA-induced hamster buccal squamous cell carcinoma burden was lessened when TQ was administered by gavage (Rajkamal et al., 2010). A significant attenuation has been reported in breast, gastric, and colon cancer xenografts after intra peritoneal administration of TQ (Gali-Muhtasib et al., 2008; Lei et al., 2012; Woo et al., 2013). TQ, when administered intraperitoneally suppressed the formation of aberrant crypt foci and colon adenoma burden in Balb/c mice (Gali-Muhtasib et al., 2008) and also elicited a reduction in the incidence and multiplicity of colon tumors in Wister rats both before or after treatment with 1,2-dimethyl hydrazine (Jrah-Harzallah et al., 2013). A decrease in the number of large polyps in the intestine of adenomatous polyposis coli (APC)^min+^ mice was reported by Lang et al. after oral feeding of TQ (Lang et al., 2013). The key liver enzymes namely, such as aspartate amino- transferase, alanine aminotransaminase, alkaline phosphatase, and lactate dehydrogenase exhibited reduced activity and the average number of hepatic nodules in rats treated with N-nitrosodiethylamine (NDEA) was decreased after both pre- and post-treatment with TQ. The expression of several cell proliferation markers, such as cyclin D1, cyclin E, proliferating cell nuclear antigen (PCNA), and Ki67 was also declined (Raghunandhakumar et al., 2013). A considerable decrease in the incidence and multiplicity of forestomach tumors and fibrosarcoma development in Swiss albino mice followed by treatment with benzo[α]pyrene (B[α]P) and 20-methylcholanthrene, respectively, was reported after addition of TQ in drinking water (Badary et al., 1999; Badary and Gamal El-Din, 2001). Intratumoral injection of TQ produced a marked regression in fibrosarcoma (FsaR) and squamous cell carcinomas xenograft tumors in mice (Ivankovic et al., 2006). A similar decrease was reported in prostate and lung cancer xenograft models in nude mice after subcutaneous injection of TQ (Kaseb et al., 2007; Yi et al., 2008; Jafri et al., 2010).

**Table 1:** **Chemopotentiating Role of Thymoquinone Observed in Preclinical Studies**.

**Chemotherapeutic Agents used in combination with TQ**	**Molecular mechanism of the chemotherapeutic agents**	**Identified melecular targets/pathways of TQ**	**Experimental models (Cell types)**	**Major outcome of TQ combination**	**References**
Temozolomide (TMZ)	DNA damage through alkylation and cell cycle arrest at G2/M phase	Transcrictional inhibition of autophagy promoting genes (beclin-1 and ATG-7)	*(In vitro)* GBM cancer (U87MG cell line)	Synergistic effect in cell growth inhibition and apoptosis induction	Pazhouhi et al., 2016; Khazaei and Pazhouhi, 2017;
Cisplatin	Induction of DNA damage through Pt-mediated DNA crosslinking (Alkylating-like mechanism)	Inhibition of NF-κB activation (decresed level of phosphorylated p65 in nucleus), Downregulation of pro-angiogenic factor (VEGF), oncogenic protein (c-Myc), antiapoptotic protein (Bcl-2), Chemo-protective effect	*(In vitro)* Colon cancer (COLO205, HCT116), *(In vivo)* Syngeneic mouse model of ovarian cancer (ID8-NGL cells), *(In vitro)* Cervical cancer (HeLa, SiHa), *(In vitro)* Lung cancer	Inhibition of tumorigenesis, ↓Expression of proliferation markers, Enhancement of double-strand DNA break and apoptosis, Reduced therapy-induced organ toxicity	Jafri et al., 2010; Nessa et al., 2011; Al-Malki and Sayed, 2014; Hafiza and Latifah, 2014; Wilson et al., 2015
Oxaliplatin (Oxptn)	Induction of DNA damage through Pt-mediated DNA crosslinking (Alkylating-like mechanism)	Counteracting drug resistance mechanisms	*(In vitro)* Osteosarcoma (MG63), *(In vitro)* Ovarian cancer, Pancreatic cancer	Reduced resistance to Oxptn and 5-FU and improvd cytotoxic effects at subtherpaeutic doses, Prevention of chemotherapy-induced toxicity and side effects	Banerjee et al., 2009; Nessa et al., 2011; Sarman et al., 2016
Tamoxifen (TAM)	Anti-estrogens (compete with estrogen to bind with estrogen receptor)	XIAP-mediated Akt regulation	*(In vitro)* Breast Cancer (MCF-7, MDA-MB-231)	Synergistic cytotoxic effect through apoptosis induction	Rajput et al., 2013; Ganji-Harsini et al., 2016
5-Fluorouracil (5-FU)	Antimetabolites (inhibition of DNA replication and S-phase arrest)	Repression of procancerous signaling proteins (e.g. Wnt, β-catenin, NF-κB, COX-2, iNOS, VEGF) Upregulation of anti-tumorigenic proteins (e.g. DKK-1, CDNK-1A, TGF-β1, Smad4, and GPx)	*(In vivo)* Azoxymethane-induced colorectal tumorigenesis in male Wister rats, *(In vitro)* Gastric cancer	Chemosensitization of 5-FU, Attenuation of tumorigenesis	Lei et al., 2012; Kensara et al., 2016
Gemcitabine (Gem)	Antimetabolites (nucleoside analogs)	Inhibition of NF-κB activation and Akt/mTOR/S6 signaling pathways, ↓ anti-apoptotic proteins, ↓ pro-apoptotic molecules Alteration in cancer cell metabolism by targeting pyruvate kinase M2	(*in vitro* and *in vivo*) Pancreatic cancer / (PANC-1, MIA PaCa-2)	Chemosensitization of Gem (Synergistic induction of apoptosis and tumor growth inhibition)	Banerjee et al., 2009; Mu et al., 2015
Topotecan (TP)	Topoisomerase-I inhibitor	Increased induction DNA damage and cell cycle arrest, ↓ Expression of anti-apoptotic proteins (e.g. Bcl2), ↑ Level of pro-apoptotic proteins (e.g. Bax, Caspase-3, Caspase-9)	*(In vitro)* Colorectal cancer, *(In vitro)* Acute myelogenous leukemia (U937)	Significant chemopotentiation of TP (antitumor activity at non-cytotoxic dose), Increased synergistic cytotoxic effects at non-cytotoxic dose of TP	Khalife et al., 2014, 2016
Paclitaxel (Pac)	Interfere in mitotic spindle formation through stabilization of microtubule assembly	Upregulation of tumor suppressor genes (e.g. p21, Brca1) and pro-apoptotic proteins (cleaved caspase-3 and PARP), reduction of phosphorylated p65 and Atk1	*(In vitro)* Tripple-negative Breast cancer	Synergistic inhibition of cancer cell growth along with increased cytotoxicity	Sakalar et al., 2016
Docetaxel	Microtubule disrupting agent	PI3K and ERK signaling pathways	*(In vitro)* Castrate-resistant prostate cancer (CRPC)/DU-145	Synergistic cytotoxicity and apoptosis	Dirican et al., 2015
Doxorubicin (Dox)	Anti-tumor antibiotics	Selective killing of leukemia cells, Chemo-protective effects in normal cells, Upregulation of tumor suppressor proteins (e.g. PTEN)	*(In vitro)* Acute lymphoblastic leukemia, *(In vitro)* Breast cancer, *(In vitro)* Melanoma, *(In vitro)* Colon cancer, *(In vitro)* Cervical cancer	Improved anticancer activity of Dox (↑growth inhibition and apoptosis), Less organ toxicity	Arafa el et al., 2011; Effenberger-Neidnicht and Schobert, 2011; Brown et al., 2014
Bortezomib	Proteosome inhibitor	Inhibition of NF-κB activation, ↓level of survival and angiogenic factors, Suppression of STAT3 activation	*(In vitro)* Multiple myeloma	Enhancement of overall anticancer activity (inhibition of cellular proliferation and induction of apoptosis)	Li et al., 2010; Siveen et al., 2014
miR-34a	microRNA	Targets various epithelial to mesenchymal transition-inducing transcription factors (EMT-TFs) including twist-related protein 1 (TWIST1), SLUG, and NOTCH1	*(In vivo)* Human metastatic breast cancer cells	Co-delivery of TQ and miR-34a resulted in synergistic inhibition of metastatic signaling pathways	Imani et al., 2017

Pazhouhi et al. demonstrated that TQ synergistically augmented the anti-cancer activity of temozolomide (TMZ) in the glioblastoma (GBM) cell line U87MG through the inhibition of autophagy (Pazhouhi et al., 2016). Another recent study by Khazaei et al. reported synergistic apoptotic cell death of glioblastoma cells upon combination treatment with TQ and TMZ (Khazaei and Pazhouhi, 2017). Similar finding was also reported by Gurung et al. where TQ was shown to inhibit proliferation and induce DNA damage, cell cycle arrest and apoptosis in the glioblastoma cells (Gurung et al., 2010). This group of researchers demonstrated that through the inhibition of telomerase activity TQ was able to promote telomere attrition in GBM cells and the effect was more pronounced in GBM cells that abundantly expressed DNA-PKcs (Gurung et al., 2010). There is also a report where TQ was found to restrain the tumor growth and enhance the chemopreventive effect of 5-fluorouracil in an early colorectal tumor model in rat (Kensara et al., 2016). In this model azoxymethane (AOM) was used to induce colorectal neoplasia and treatment with 5-FU/TQ combination produced a greater reduction of AOM-induced colorectal tumors and large aberrant crypts foci compared to either agents alone. TQ collaborated with 5-FU to inhibit the expression of procancerous *NF-*κ*B*, iNOS, VEGF, *Wnt*, β*-catenin, COX-2*, and TBRAS with a concomitant increase in anti-tumorigenic *TGF-*β1, TGF-βRII, *DKK-1, CDNK-1A, Smad4*, and GPx expression (Kensara et al., 2016). They also concluded with no significant differences in the liver function enzymes and renal function parameters between the control and treatment groups, demonstrating an excellent safety profile of the TQ/5-FU combination therapy (Kensara et al., 2016). A recent study by Fröhlich et al. demonstrated enhanced level of ROS generation and concomitant DNA damage induction in human colon cancer cells treated with a novel hybrid of TQ and artemisinin (Frohlich et al., 2017). In another study, conducted by Lei et al., TQ was found to chemosensitize 5-FU in gastric cancer treatment through increased apoptosis induction and growth inhibition (Lei et al., 2012).

A study conducted by Siveen et al. demonstrated that TQ was able to inhibit multiple myeloma (MM) cell proliferation along with induction of chemosensitization in xenograft mouse model (Siveen et al., 2014). TQ itself inhibited the proliferation of MM cells and CD138+ cells isolated from MM patient samples in a concentration dependent manner. TQ treatment elicited a potentiation of apoptosis exerted by bortezomib as evidenced by the activation of caspase-3 and cleaved PARP (Siveen et al., 2014). Most importantly, the antitumor efficacy of bortezomib in a xenograft model was potentiated by TQ through the modulation of different survival and angiogenesis markers such as Bcl-2, p65, Ki-67, and VEGF (Siveen et al., 2014). A similar study was done by Li et al. on MM model with constitutively activated signal transducer and activator of transcription 3 (STAT3) pathway, where TQ treatment resulted in suppression of STAT3 phosphorylation/activation followed by significant potentiation of thalidomide and bortezomib efficacy, in terms of apoptosis induction and growth arrest in MM cells (Li et al., 2010).

TQ also displayed promising results both *in vitro* and *in vivo* in an orthotopic model of pancreatic cancer. There was a significant growth inhibition of tumor cells when TQ was added to gemcitabine or oxaliplatin (Banerjee et al., 2009; Mu et al., 2015). This was accompanied by an increase in tumor cell killing through the down-regulation of NF-κB, Bcl-2, survivin, and cyclooxygenase-2. Concomitantly, an enhanced apoptosis and diminished proliferation of the tumor tissues were supportive of strong chemosensitization potential of TQ in the orthotopic pancreatic mouse model. Synergistic combinations of TQ and oxaliplatin were also investigated by some other groups where a third chemotherapy (5-FU or cisplatin) was used along with TQ and oxaliplatin, resulted in reversal of chemo resistance and improved cytotoxic effects at sub-therapeutic doses of chemo agents (Nessa et al., 2011; Sarman et al., 2016). Moreover, there combinations were found to exhibit chemo-preventive effects and reduced chemotherapy-induced toxicities and side effects. Several other preclinical studies have reported synergistic effect of TQ and cisplatin on multiple *in vitro* and *in vivo* cancer models including colon, ovarian, cervical, and lung cancer (Jafri et al., 2010; Nessa et al., 2011; Al-Malki and Sayed, 2014; Hafiza and Latifah, 2014; Wilson et al., 2015). In these studies, TQ was found to downregulate pro-angiogenic factors (e.g., VEGF), growth promoting oncogenic proteins (e.g., cMyc), and anti-apoptotic proteins (e.g., Bcl-2) through inhibition of NF-κB activation. Combination treatment with TQ and cisplatin showed hindrance in tumorigenesis evident from diminished expression of proliferation markers, a concomitant enhancement of double strand DNA break and apoptotic cell death were also observed (Jafri et al., 2010; Wilson et al., 2015).

TQ treatment also showed promising results in doxorubicin-resistant human breast cancer cells (Arafa el et al., 2011). After doxorubicin-resistant MCF-7/DOX cells were exposed to TQ, there was an extensive decrease of the cell survival regulators, phosphorylated Akt and Bcl2 along with an increased expression of PTEN and apoptotic markers such as Bax, cleaved caspases, and cleaved PARP. TQ also produced an augmented expression of p53 and p21 proteins with a concomitant G2/M arrest in the same cell line (Arafa el et al., 2011). TQ-mediated chemopotentiation of doxorubicin was also observed in several other cancer models including acute lymphoblastic leukemia, melanoma, colon cancer, and cervical cancer (Effenberger-Neidnicht and Schobert, 2011; Brown et al., 2014). In addition to the induction of synergistic cytotoxic effects through interference in tumor growth and survival signaling, combination treatment with TQ also exhibited chemo-protective effects and limited organ toxicity (Brown et al., 2014). Numerous other studies have investigated the potential chemo-potentiating effects of TQ with diverse classes of chemotherapeutic drugs (summarized in Table 1) including anti-estrogen (e.g., tamoxifen), topoisomerase-I inhibitor (e.g., topotecan), and microtubule disrupting agents (Rajput et al., 2013; Khalife et al., 2014, 2016; Dirican et al., 2015; Ganji-Harsini et al., 2016; Sakalar et al., 2016). Rajput et al. recently reported that TQ can cause reversal of tamoxifen resistance in triple negative breast cancer cells by interfering in Akt-mediated induction of apoptosis inhibitory protein such as X-linked inhibitor of apoptosis protein (XIAP; Rajput et al., 2013). Chemo-potentiation of microtubule disrupting agents (e.g., docetaxel and paclitaxel) by TQ was found to be mediated by upregulation of tumor suppressor genes such as p21 and Brca1, induction of pro-apoptotic factors, and inhibition of cancer cell growth and survival promoting signaling pathways such as PI3K/Akt and MAPK/ERK (Dirican et al., 2015; Sakalar et al., 2016).

Most of the above mentioned studies have observed synergistic cytotoxic and other anti-tumorigenic effects (e.g., angiogenesis, migration, invasion, and metastasis) of TQ against cancer cells with simultaneous protection of non-cancerous cells from chemotherapy induced hazardous effects, through preferential modulation of a complex array of tumorigenic, and deregulated signaling pathways. In addition to these chemopotentiating effect, TQ is recently found to synergize with miR-34a, a microRNA which targets various epithelial to mesenchymal transition-inducing transcription factors (EMT-TFs) including twist-related protein 1 (TWIST1), SLUG, and NOTCH1 (Imani et al., 2017). Overall, TQ was found to show almost all the favorable characteristics of an ideal adjuvant agents that can be used along with a wide variety of main course anticancer chemotherapeutic drugs. These emerging evidences justify further in-depth evaluation of TQ's chemopotentiating role in preclinical and clinical level.

## Immunomodulatory effects of TQ and its prospective use with immunotherapies

Possible immunomodulatory role of TQ is evident from numerous *in vitro* and *in vivo* studies (Gholamnezhad et al., 2015; Majdalawieh and Fayyad, 2015), where TQ was found to regulate the growth and cellular response of various immune cells such as T cells, B cells, macrophages, neutrophils, NK cells, and dendritic cells. Beside other inflammatory mediators, these immune cells are essential components of the tumor microenvironment and frequently release different cytokines and growth factors that lead to the generation of immunosuppressive milieu at the tumor sites along with promotion of cancer cell proliferation, survival, migration, invasion, and metastasis (Lin and Karin, 2007). During tumorigenesis, a pre-existing chronic inflammatory conditions elicit higher level of immune inhibitory cytokines and other immunosuppressive factors. Likewise, the immune cells infiltrated in the tumor microenvironment such as cytotoxic T cells and natural killer (NK) cells, rather showing tumoricidal activity, feed into the tumor associated inflammation (Zou, 2005). Due to this dysregulated immune response, immunomodulatory drugs are now considered as potential supplementary agents with various immunotherapies including cancer vaccines, immune checkpoint blocking antibodies, adoptive T cell therapy, and dendritic cell (DC) based immunotherapy (Mahoney et al., 2015). Moreover, immunomodulatory agents can functionalize cancer related flawed innate and adaptive immune systems to enhance anticancer immune activity.

Several lines of studies have confirmed that TQ can exert anti-inflammatory effect through inhibition of eicosanoids and prostaglandine synthesis (Houghton et al., 1995; El Mezayen et al., 2006) and intervention in the production and release of pro-inflammatory cytokines and reactive oxygen and nitrogen species (Mansour et al., 2002; Sankaranarayanan and Pari, 2011; Umar et al., 2012). A direct inhibitory effect of TQ treatment on NF-κB activation was also reported in multiple occasions (Mohamed et al., 2005; El Gazzar et al., 2007; Sethi et al., 2008; Zhang et al., 2016), which might explain the downregulation of pro-inflammatory mediators upon TQ administration. Although, NF-κB-mediated gene transcription is required for normal cellular activities, most of the cancers are associated with aberrant NF-κB signaling that lead to the release of different inflammatory cytokines followed by activation of diverse arrays of tumorigenic signaling pathways (Karin, 2006, 2009). Therefore, by attenuating NF-κB-mediated gene transcriptions, TQ might alter inflammation-induced immunosuppression in tumor microenvironment, as well as can restrict the tumorigenesis processes. Signal transducer and activator of transcription 3 (STAT3) is a key player in the mechanism of tumor-induced immune deregulation characterized by the paucity of immunological danger signals essential for immune activation and escaping of cancer cells from natural immune surveillance (Yu et al., 2007). Constitutive activation of STAT3 has been observed in many cancers which results in the generation of immature myeloid and dendritic cells. The subsequent prevention of DC maturation leads to immune tolerance due to T cells deletion or their differentiation into regulatory or suppressor T cells. In addition, persistent activation of STAT3 in NK cells and neutrophils inhibit the tumor killing activity of those effector cells. A study by Li et al. reported that TQ can interfere in both the constitutive and IL-6-inducible STAT3 phosphorylation through inhibition of upstream signaling kinases (Li et al., 2010). Similar observations were stated by couple of other groups where TQ treatment resulted in decease phosphorylation and subsequent activation of STAT3 (Badr et al., 2011c; Kundu et al., 2014a). Though, there are no reports showing the correlation of TQ-mediated suppression of STAT3 activation and reversal of immune dysregulation, several studies have investigated the effect of TQ treatment on DC maturation (Xuan et al., 2010), cytotoxic T cell activation (Badr et al., 2011a; Salem et al., 2011), and enhancement of NK cytotoxic functions (Salim et al., 2014).

Treating cancer with immunotherapies have gained immense interest, mostly due its unique feature of re-weoponizing body's own immune systems to identify and destroy tumor cells. After long history of failure of immunotherapy, time has now changed and progress has been made for effective cancer immunotherapy against certain cancers especially after successful introduction of immune checkpoint blocking antibodies, e.g., cytotoxic T lymphocyte associated protein-4 (CTLA-4) and program cell death protein-1 (PD-1) blocking antibodies. However, there are some other strategies which can be utilized as successful cancer immunotherapies including cancer vaccines, adoptive T cell therapy and DC based immunotherapy. Effect of TQ in some of the cases has been shown by some research groups whereas there are still lots of research yet to be done in this specific field. Cancer vaccination is a promising approach toward cancer immunotherapy. An effective anti-tumor response is obtained if antigen is captured and processed by dendritic cells, presented through its MHC molecule to CD4/CD8 T cells, and subsequently activation and proliferation of T cells occurred until it eliminates the cancer cell (Mellman et al., 2011). Suppression at any point can cause immune tolerance. In order to make a successful cancer vaccine, it needs to break the tolerance obtained against tumor cells (Mellman et al., 2011; Topalian et al., 2011; Farkona et al., 2016). Among different categories of cancer vaccines, peptide vaccines, tumor and immune cell vaccines are very crucial in cancer immunotherapy (Subramaniam et al., 2016). Tacemotide, a peptide vaccine that target MUC1 glycoprotein is one of the prominent vaccine in this group whereas Sipuleucel-T is an immune cell vaccine in prostate cancer (Anassi and Ndefo, 2011; Wurz et al., 2014; Hossain and Wall, 2016).

Dendritic cells, because of its antigen presenting abilities and role in correlation between innate and adaptive immunity, can be utilized to target large number of antigens and activate in order to break the tolerance (Palucka and Banchereau, 2012). The target of vaccination with DC is to see if it can induce cytotoxic CD8+ effector T cells which are tumor specific (Palucka and Banchereau, 2012). While immature dendritic cells are able to capture and process antigen, it needs to be matured in order to present antigenic peptide to naïve T cells in the lymphoid organ. The most common method to mature DC is cytokine cocktail that includes TNFα with combination of other cytokine, e.g., IL6, PGE2 (Bol et al., 2016). Other factors include bacterial components such as lipopolysaccharides (LPS), CD40 ligand, IFNα and IFNγ (Castiello et al., 2011). One group has investigated if TQ has any role on LPS induced maturation of DC and cytokine release (Xuan et al., 2010). They found out that TQ inhibits maturation and impairs cytokine release by LPS stimulated DCs. Instead, it stimulates DC apoptosis by caspase activation and impairing the phosphorylation of LPS induced Akt and ERK1/2. However, there are still research needs to be done to see if TQ helps in DC maturation in presence of different cytokines, an important aspects for DC based immunotherapy.

T cell based immunotherapy mostly includes adoptive T cell transfer and immune checkpoint inhibitor antibodies (Houot et al., 2015). Adoptive cell therapy emerged as one of the most successful cancer immunotherapy in metastatic melanoma cancer patients (Rosenberg et al., 2008). It is being used to artificially enrich the quality and quantity of T lymphocytes that can detect tumor specific antigen and kill tumor. This process requires to harvest patients own T lymphocytes either from peripheral blood or draining lymph node, expand *in-vitro* with the stimulation of anti-CD3 and anti-CD28 monoclonal antibodies in presence of IL-2 and reintroduce back into the patient's body (Houot et al., 2015). Those adoptively transferred T cells, because of their specificity to tumor antigen, can recognize the tumor antigen that is being presented through MHC1 complex and improve cell engraftment and survival (Salem et al., 2011). Those functionally significant anti-tumor T cells can be recognized by the co expression of lymphoid homing molecule L-selectin CD62L^+^ and chemokine receptor CCR7^+^ (Rosenberg et al., 2008; Klebanoff et al., 2012). It has been shown that TQ can increase the survival rate of CD62L at a lower dose. It also increased CD8^+^ T cell proliferation and the production of CD8+ released cytokine IFN-γ, meaning it can enhance the survival of antigen stimulated CD8^+^ T cells. However, high dose of TQ might cause toxic effect and leads to apoptosis of T cells (Salem et al., 2011).

Over-all, immunomodulatory activity of TQ, pointed by experimental evidences, can be exploited by combining it with various immunotherapies such as monoclonal antibodies, immune checkpoint inhibitors, and cancer vaccines (**1**). More studies are warranted to investigate the feasibility of priming the innate and adaptive immune systems by TQ and its potential to improve immunotherapeutic efficacy.

## Summary and future perspective

Though considerable advancements have been accomplished in cancer pathogenesis and thereby in treatment strategies, the overall survival rates still remain poor. Targeting a particular molecule or signaling pathway, involved in one of the singular aspects of the multistep complex tumorigenesis processes, has recently been deemed as extravagant attempt to curtail malignant progression. Due to the inherent heterogeneous nature, some cancer cells can always evade a particular therapeutic modality and continue to survive on alternative pathways followed by recurrence of tumor at a far more aggressive form. Therefore, the paradigm in cancer treatment strategy is now shifting from targeted therapy to combination or multi-targeted approaches.

TQ, immediately after its isolation, has been investigated in numerous disease models including cancer. Those studies have identified some important and useful pharmacological properties of TQ that can be exploited to devise novel and more effective therapeutic interventions against cancer. It was found to exhibit a wide range of biochemical functions through its modulatory interactions with diversified molecular targets. Reportedly, TQ interferes in the phosphorylation and subsequent activation of several upstream tyrosine kinases (e.g., MAPK, Akt, mTOR, PIP3) that are involved in tumor cell proliferation signaling pathways (Yi et al., 2008; Kundu et al., 2014b). Transcriptional factors (e.g., Nrf2, NF-κB, and STAT-3), key players in various oncogenesis process, are other crucial molecular targets of TQ (Kundu et al., 2014b; Darakhshan et al., 2015; Majdalawieh et al., 2017). By regulating the activation of these transcription factors, TQ can counteract different tumorigenic processes including inflammation, cell proliferation, cell survival, angiogenesis, cell invasions, and metastasis. Moreover, TQ shows chemopreventive properties by downregulating carcinogen metabolizing enzymes (e.g., CYP 1A2, CYP 3A4), upregulating cytoprotective enzymes (e.g., glutathione S-transferase, superoxide dismutase, and oxidoreductase), attenuated production of pro-inflammatory mediators (e.g., cytokines, chemokines, and prostaglandins; Kundu et al., 2014b).

By virtue of its multi-targeting nature, TQ can be considered as a promising therapeutic moiety for cancer treatment. But the main focus of this review was to show how TQ can improve the therapeutic efficacy and safety profile of the main course cancer therapies, namely surgery, radiotherapy, chemotherapy, and immunotherapy through induction of selective cytotoxicity in cancer cells and cytoprotection in healthy cells. This was primarily because of the fact that TQ has low potency and poor bioavailability. These issues can be resolved by synthesizing various analogs of TQ and formulating those into different delivery systems. We have to also address some other critical questions about TQ regarding its opposite biochemical functions, such as it can act both as an antioxidant (at low concentration) and ROS inducer (at relatively high concentration). To better understand these differential cellular effects of TQ, more *in vitro, in vivo*, and *in silico* studies should be conducted at both proteomic and genomic level. Furthermore, upcoming studies should be concentrated on finding better derivatives of TQ along with detailed and accurate characterizations thereof. We have to also come up with suitable dosage form and delivery system for those analogs along with determination of their pharmacokinetic behavior, efficacy, and toxicity in multiple *in vivo* cancer models. Findings from such studies will enable us to devise clinically effective combination therapeutics where TQ or its derivatives can potentiate the anti-tumorigenic potential of various conventional and established anti-cancer agents.

## Author contributions

AM wrote the major portion of the manuscript and coordinated the manuscript writing, MH wrote the “Immunomodulatory effects of TQ and its prospective use with immunotherapies” portion of the manuscript; DB wrote the “Chemo-potentiating role of TQ” portion of the manuscript and MB conceived the idea and contributed writing and coordinating the manuscript.

### Conflict of interest statement

The authors declare that the research was conducted in the absence of any commercial or financial relationships that could be construed as a potential conflict of interest.
